# Gender Difference in the Prevalence of Insomnia: A Meta-Analysis of Observational Studies

**DOI:** 10.3389/fpsyt.2020.577429

**Published:** 2020-11-20

**Authors:** Liang-Nan Zeng, Qian-Qian Zong, Yuan Yang, Ling Zhang, Yi-Fan Xiang, Chee H. Ng, Li-Gang Chen, Yu-Tao Xiang

**Affiliations:** ^1^Department of Neurosurgery, The Affiliated Hospital of Southwest Medical University, Luzhou, China; ^2^Neurosurgery Clinical Medical Research Center of Sichuan Province, Academician (Expert) Workstation of Sichuan Province, Luzhou, China; ^3^Unit of Psychiatry, Faculty of Health Sciences, Institute of Translational Medicine, University of Macau, Macao, China; ^4^Center for Cognition and Brain Sciences, University of Macau, Macao, China; ^5^Institute of Advanced Studies in Humanities and Social Sciences, University of Macau, Macao SAR, China; ^6^The National Clinical Research Center for Mental Disorders & Beijing Key Laboratory of Mental Disorders, Beijing Anding Hospital & the Advanced Innovation Center for Human Brain Protection, Capital Medical University, Beijing, China; ^7^Pui Ching Middle School Macau, Macao, China; ^8^Department of Psychiatry, The Melbourne Clinic and St. Vincent's Hospital, University of Melbourne, Melbourne, VIC, Australia

**Keywords:** insomnia, prevalence, gender difference, meta-analysis, observational studies

## Abstract

**Objective:** Insomnia is a major health challenge in the general population, but the results of the gender differences in the epidemiology of insomnia have been mixed. This is a meta-analysis to examine the gender difference in the prevalence of insomnia among the general population.

**Methods:**Two reviewers independently searched relevant publications in PubMed, EMBASE, PsycINFO, Web of Science from their inception to 16 April 2019. Studies that reported the gender-based prevalence of insomnia according to the international diagnostic criteria were included for analyses using the random-effects model.

**Results:**Eventually 13 articles were included in the meta-analysis. The pooled prevalence of insomnia in the general population was 22.0% [*n* = 22,980, 95% confidence interval (CI): 17.0–28.0%], and females had a significantly higher prevalence of insomnia compared with males (OR = 1.58, 95% CI: 1.35, 1.85, *Z* = 5.63, *p* < 0.0001). Subgroup analyses showed that greater gender difference was associated with the use of case-control study design and consecutive sampling method. Meta-regression analyses also revealed that higher proportion of females and better study quality were significantly associated with greater gender difference.

**Conclusions:**This meta-analysis found that the prevalence of insomnia in females was significantly higher than males in the included studies. Due to the negative effects of insomnia on health, regular screening, and effective interventions should be implemented in the general population particularly for females.

## Introduction

As a major health challenge in the general population, the prevalence of insomnia has been rising over time globally (1). Insomnia is characterized by difficulty in initiating or maintaining sleep (2, 3). According to the Diagnostic and Statistical Manual of Mental Disorders (DSM−5), insomnia is defined by difficulty in falling asleep, staying asleep or early morning awakenings more than 3 times per week for more than 3 months, and is associated with subjective poor sleep quality, as well as daytime dysfunction (4). Several diagnostic criteria for insomnia have been widely used in clinical practice, including the International Classification of Sleep Disorder (ICSD), the DSM, and International Classification of Diseases (ICD) systems (e.g., codes 307.41, 307.42, or 780.52 in the ICD-9) (4–9). In addition, certain standardized diagnostic instruments for insomnia based on the ICD/DSM criteria have been developed, such as the World Health Organization Composite International Diagnostic Interview (CIDI) (10–13).

Due to its chronic nature, insomnia usually leads to many negative outcomes, such as impaired daily functioning, lower quality of life, and poor mental health (3, 14, 15). For instance, insomnia commonly occurs with psychiatric disorders, especially depression, anxiety, and psychosis (16, 17). In order to reduce its adverse outcomes and allocate appropriate health resources, health professionals and policymakers need to understand the patterns and clinical features of insomnia. Prevalence of insomnia in the general population varies greatly across studies, ranging from 5.8 to 32.8% (18–21), and is associated with certain demographic factors, such as age, gender, marital status, income, and education level (22).

Gender differences in insomnia and related health issues have been widely examined (23–27). Such differences are not only associated with the etiological and pathophysiological processes, but also associated with the treatments and prognosis of insomnia (24). The results of gender differences in the prevalence studies of insomnia have been mixed. Some studies found that women suffered from insomnia more frequently than men (8, 18, 19), while opposite findings were found in other studies (28). A previous meta-analysis of cross-sectional studies (29) found that women had a higher risk of insomnia [risk ratio (RR) = 1.41]. However, RR was used as the effect size in this study, which is a major limitation. Odds ratio (OR) is mainly used for cross-sectional studies, while RR is used for cohort studies, both of which are comparable in magnitude only when the prevalence of the target disease is rare, such as cancer (30, 31). In addition, sophisticated analyses, such as subgroup and meta-regression analyses, were not performed, and publication bias and quality assessment were not tested. Furthermore, studies using both international diagnostic criteria and screening scales for insomnia were included, even though the prevalence of insomnia in studies based on diagnostic criteria was significantly lower than those using screening scales (32). Due to the abovementioned limitations of the previous meta-analysis and considering newly published studies on gender difference since then we conducted this meta-analysis to examine the gender difference in the prevalence of insomnia in the general population as defined by international diagnostic criteria.

## Methods

### Literature Search

This meta-analysis was conducted based on the Preferred Reporting Items for Systematic Reviews and Meta-Analyses (PRISMA). A systematic search was independently performed by two reviewers (LNZ and QQZ) in the following databases: PubMed, EMBASE, PsycINFO, Web of Science from their date of inception to 16 April 2019, using the following search terms: insomnia, sleepless, quality of sleep, dyssomnia, sleep complaint, sleep problem, sleep disturbance, sleep disorder, Diagnostic and Statistical Manual of Mental Disorders, DSM, International Classification of Sleep Disorder, ICSD, International Classification of Diseases, ICD, survey, cross-sectional, prevalence, and epidemiology.

### Study Selection and Data Extraction

Cross-sectional, case-control and cohort studies (only the baseline data were extracted in cohort studies) which fulfilled the following inclusion criteria were included: (1) stratified prevalence of insomnia by gender; (2) insomnia diagnosed by international diagnostic criteria, such as the DSM, ICSD, and ICD systems; (3) publications in English. Studies conducted in special groups, such as sleep medication users and militants, were excluded. The study selection and data extraction were independently performed by the same two reviewers (LNZ and QQZ). Any disagreements between the two reviewers were resolved by involving a third reviewer (YTX). The titles and abstracts were first reviewed by the two reviewers independently, and then the full text of potentially relevant articles were examined for eligibility (33). If more than one article were published based on the same dataset, only the article with the largest simple size was included. Information of study characteristics (i.e., the first author, publication year, year of survey, country, continent, study design, sample size, response rate, and sampling method), population characteristics (i.e., age, female proportion), insomnia measures (i.e., diagnostic and type of interview) and the prevalence of insomnia (i.e., prevalence and prevalence in male and female groups) were extracted for analyses.

### Quality Evaluation

As recommended previously (34–36), the quality evaluation was assessed by two reviewers (LNZ and QQZ) using an instrument with 6 items on the methodological quality of observational studies including sampling method, response rate, the representativeness and definition of targeted population, definition of insomnia, and validation of assessment instrument of insomnia.

### Statistical Analyses

Stata version 12.0 and Comprehensive Meta-Analysis statistical software Version 2.0 were used to pool data using the random effects model (37). Heterogeneity between studies was assessed using *I*^2^ statistic (*I*^2^ > 50% considered substantial heterogeneity) (38). The funnel plot and Egger's-test were used to test publication bias (39). Sensitivity analysis was performed by excluding each study individually. All the statistical significance level was set at *P* < 0.05 (two-sided).

Subgroup and meta-regression analyses were carried out to explore possible sources of heterogeneity. The following categorical variables were explored: (1) year of survey: 1990–2004 vs. 2007–2012, using median splitting method; (2) study design: case-control vs. cohort vs. cross-sectional; (3) sampling method: consecutive vs. multi-stage stratified vs. random sampling; (4) type of interview: face to face vs. telephone interview; (5) age: ≤ 44.7 vs. >44.7 years, using splitting method; (6) continent: Africa vs. Asia vs. Europe vs. North America. Meta-regression analyses were conducted to examine the moderating effects of the proportion of females and study quality on the results.

## Results

### Characteristics of Included Studies and Quality Assessment

**Figure 1:**
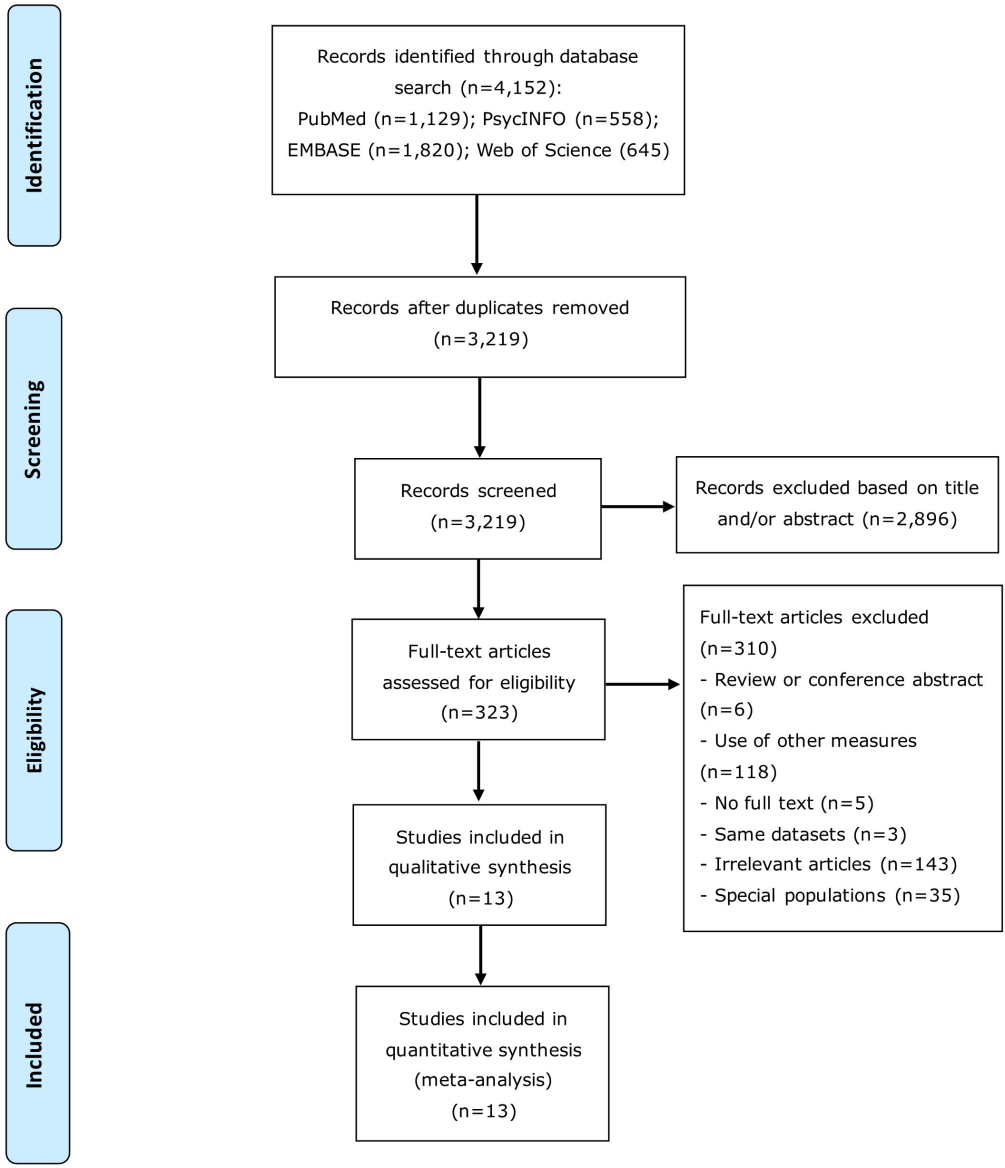
Flowchart of study selection.

Initially, a total of 4,152 articles were identified. After screening titles or/and abstract, 323 articles were further reviewed and eventually 13 articles were included for analysis. The procedure of the literature search is presented in Figure 1. The 13 studies covered 326,908 participants in total (139,349 females and 187,559 males; Table 1). The studies were published between 1994 and 2017, and the sample size ranged from 290 to 299,188, with the response rate ranging from 33.4 to 97.5%.

**Table 1:** Characteristics of studies included in the meta-analysis.

**No**.	**First author (publication year)**	**References**	**Country**	**Continent**	**Year of survey**	**Study design**	**Sample size**	**Sampling method**	**Diagnostic classification**	**Age (mean)**	**Female proportion (%)**	**Type of interview**	**Quality score**
1	Kim (2017)	(19)	Korea	Asia	NR	Cross-sectional	881	Random	ICSD-2	70.6	59.0	Face-to-face interview	5
2	Chiou (2016)	(18)	China (Taiwan)	Asia	2002	Cross-sectional	4,047	Cluster	DSM-IV	NR	44.2	Face-to-face interview	4
3	Barclay (2015)	(40)	US	North America	1990	Cohort	2,822	NR	DSM-III-R	NR	54.0	Semi-structured interview	4
4	Hysing (2013)	(20)	Norway	Europe	2012	Cross-sectional	9,846	NR	DSM-IV and DSM-V^&^	17.0	53.0	NR	4
5	López-Torres (2012)	(28)	Spain	Europe	2009	Cross-sectional	926	Random	DSM-IV-TR	74.4	54.2	Face-to-face interview	5
6	Morin (2011)	(21)	Canada	North America	2007	Cross-sectional	2,000	Random	DSM-IV-TR and ICD-10	48.6	60.5	Telephone interview	5
7	Kleinman (2009)	(41)	US	North America	2007	Case-control	299,188	NR	ICD-9	40.2	41.2	NR	3
8	Gureje (2009)	(10)	Nigeria	Africa	2004	cohort	2,152	Multi-stage stratified	CIDI-3	NR	53.8	Face-to-face interview	5
9	López-Torres (2007)	(42)	Spain	Europe	NR	Cross-sectional	424	NR	DSM-IV	NR	58.3	NR	5
10	Morin (2006)	(43)	Canada	North America	2002	Cross-sectional	2,001	Random	DSM-IV and ICD-10	44.7	58.0	Telephone interview	5
11	Pallesen (2001)	(44)	Norway	Europe	NR	Cross-sectional	2,001	Random	DSM-IV	44.7	54.5	Telephone interview	5
12	Canals (1997)	(45)	Spain	Europe	NR	Cross-sectional	290	NR	DSM-III-R and ICD-10^*^	18	52.4	Face-to-face interview	4
13	Hohagen (1994)	(46)	Germany	Europe	NR	Cross-sectional	330	Consecutive	DSM-III-R	74.0	72.0	NR	5

*NR, Not reported; DSM, Diagnostic and Statistical Manual of Mental Disorders; ICSD, International Classification of Sleep Disorder; ICD, International Classification of Diseases*.

& *Only insomnia diagnosed by DSM-IV was used for analyses*.

* *The prevalence of insomnia diagnosed by DSM-III-R and ICD-10 was the same and the DSM-III-R was used for analyses*.

The mean score of the study quality assessment was 4.54, ranging from 3 to 5. In all studies, insomnia was defined by validated instruments. The population sample of one study was not defined clearly; the samples of 6 studies were not recruited by random or consecutive sampling methods; the response rates of 10 studies were <70%; and the samples of 2 studies were not representative.

### The Overall Prevalence of Insomnia and Gender Difference

**Figure 2:**
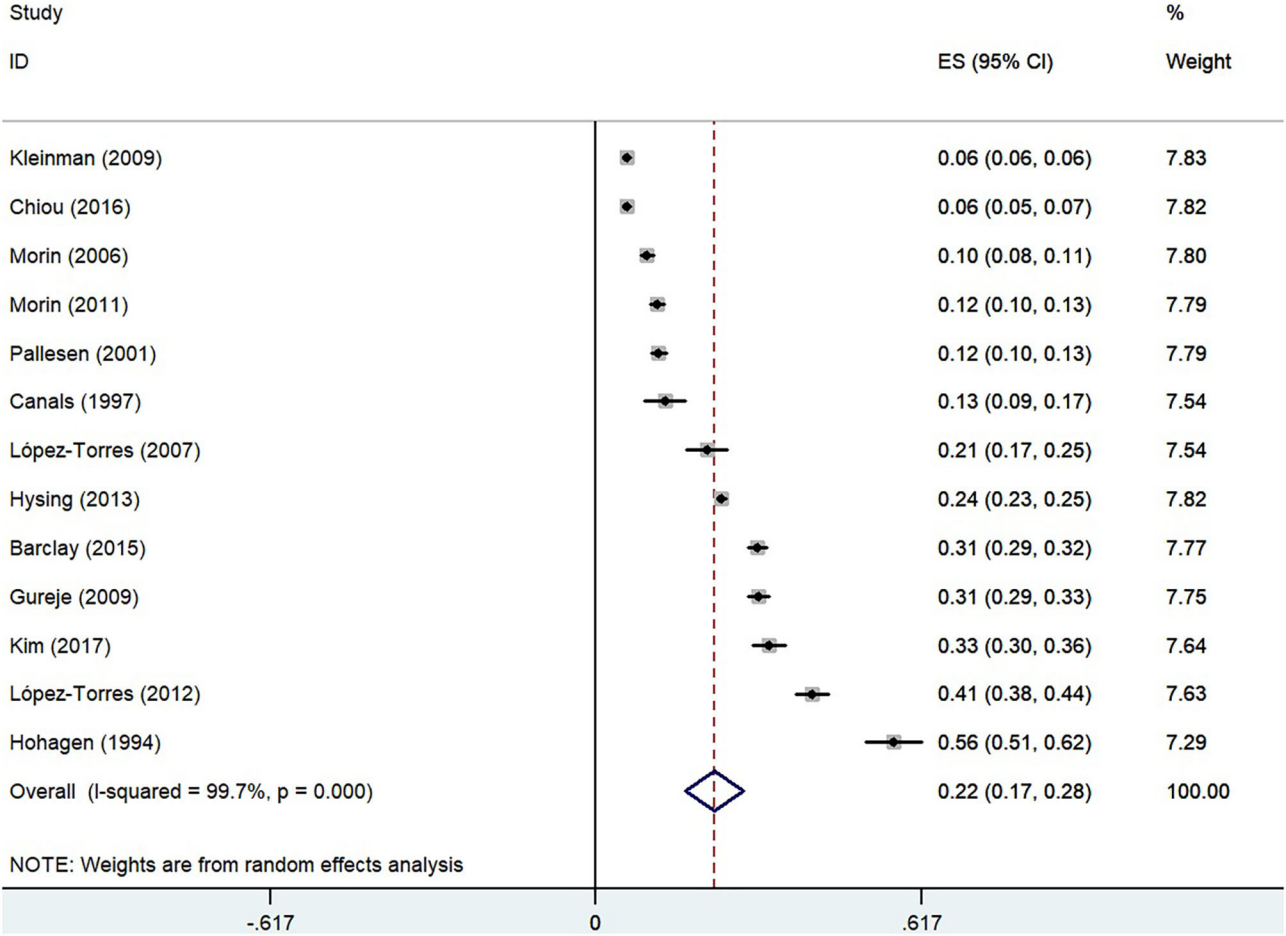
Forest plot of the prevalence of insomnia in the general population.

The pooled overall prevalence of insomnia using the random-effect model was 22.0% [*n* = 22,980, 95% confidence interval (CI): 17.0–28.0%, *I*^2^ = 99.7%], as shown in Figure 2.

**Figure 3:**
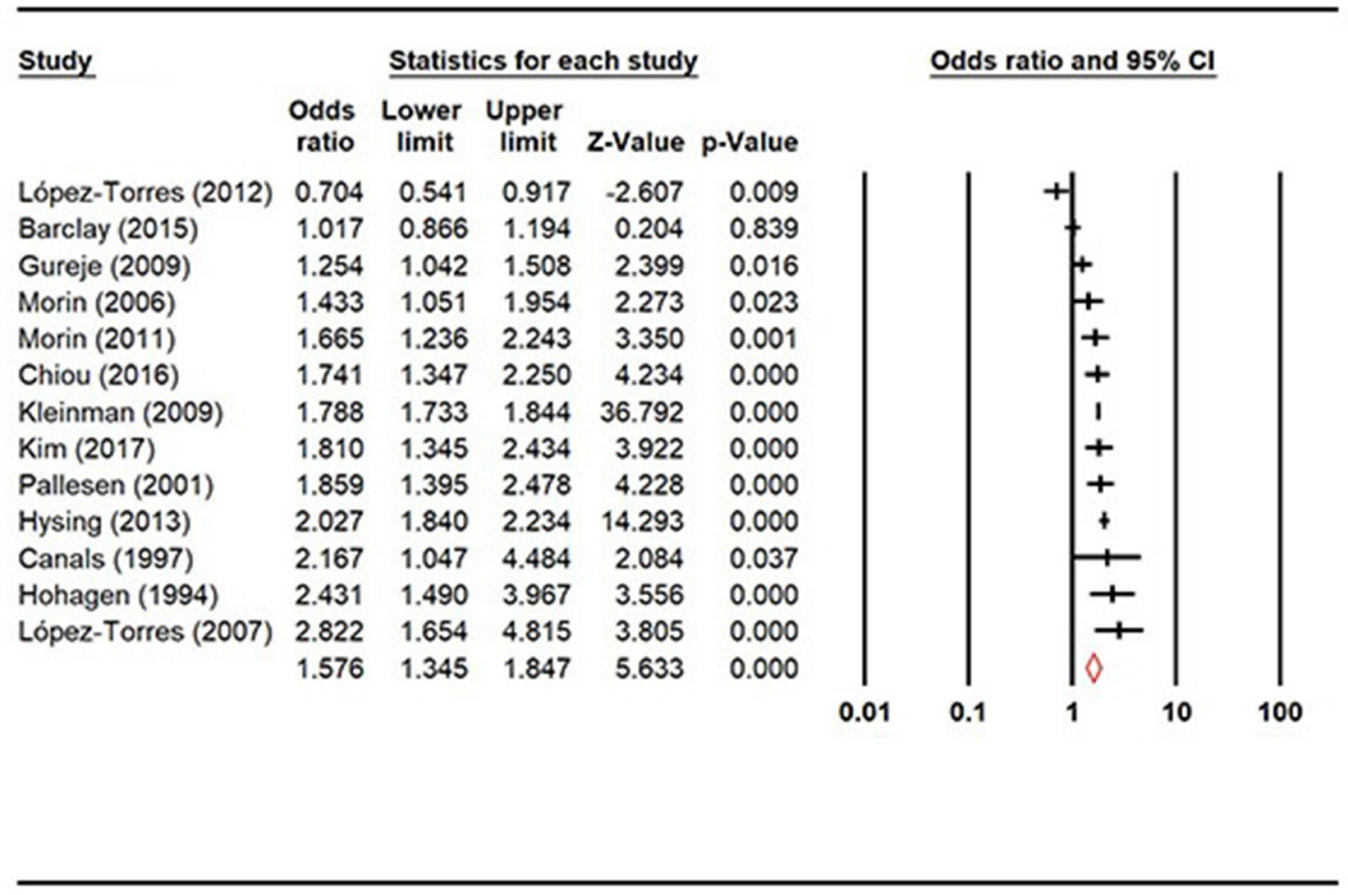
Forest plot of gender difference in the prevalence of insomnia.

Prevalence of insomnia was compared between female and male populations (Figure 3), and the result showed that females had significantly higher prevalence of insomnia than males (OR = 1.58, 95% CI: 1.35, 1.85, *Z* = 5.63, *p* < 0.0001).

### Subgroup Analyses and Meta-Regression

Subgroup analyses (Table 2) revealed that greater gender difference was significantly associated with the use of case-control study design and consecutive sampling method. In contrast, the gender difference was not associated with year of survey, type of interview, age, or continent (all *p*-values > 0.05).

**Table 2:** Subgroup analyses between female and male groups.

**Subgroups**	**Categories (No. of studies)**	**Sample size**		**OR**	**95% CI (%)**		***I^**2**^* (%)**	***P*-value within subgroup**	**Q (*P*-value across subgroups)**
		**Female**	**Male**		**Lower**	**Upper**			
Year of survey (years)	1990–2004 (4)	5,625	5,397	1.23	1.11	1.37	77.60	0.004	0.51 (0.48)
	2007–2012 (4)	131,447	180,483	1.79	1.74	1.84	94.52	<0.001	
Study design	Case-control (1)	124,550	174,638	1.79	1.73	1.84	<0.001	1.00	19.39 **(<0.001)**
	Cohort (2)	2,681	2,239	1.11	0.99	1.26	64.41	0.09	
	Cross-sectional (10)	12,118	10,628	1.79	1.67	1.93	85.34	<0.001	
Sampling method	Consecutive (1)	5,215	4,631	2.03	1.84	2.23	<0.001	1.00	22.55 **(<0.001)**
	Multi-stage stratified (2)	2,313	1,840	1.30	1.11	1.52	<0.001	0.47	
	Random (6)	127,171	176,444	1.77	1.72	1.82	89.90	<0.001	
Type of interview	Face to face (5)	129,520	179,464	1.73	1.68	1.78	95.67	<0.001	3.39 (0.07)
	Telephone (4)	2,119	1,382	1.85	1.54	2.23	<0.001	0.60	
Mean age (years)	≤44.7 (5)	132,164	181,162	1.86	1.80	1.91	95.01	<0.001	0.85 (0.36)
	>44.7 (4)	2,469	1,668	1.31	1.12	1.53	91.30	<0.001	
Continent	Africa (1)	1,157	995	1.25	1.04	1.51	<0.001	0.02	7.14 (0.07)
	Asia (2)	2,308	2,620	1.77	1.46	2.15	<0.001	<0.001	
	Europe (6)	7,444	6,373	1.84	1.69	2.00	91.50	<0.001	
	North America (4)	128,440	177,571	1.75	1.70	1.80	93.66	<0.001	

*Q, Cochran's Q. Bold-value: p < 0.05*.

*OR, odds ratio; CI, confidence interval*.

In the meta-regression analyses, higher proportion of females (*B* = 0.02, *Q* = 31.59, *p* < 0.001) and better study quality (*B* = 0.27, *Q* = 18.46, *p* < 0.001) were significantly associated with greater gender difference.

### Publication Bias and Sensitivity Analysis

The visual Funnel plot (Supplementary Figure 1) and Egger's-test (*t* = 1.06, *P* = 0.31) did not reveal any publication bias. Sensitivity analyses did not find any individual study that changed the significance of the primary results when each study was sequentially excluded (Supplementary Figure 2).

## Discussion

This was the first meta-analysis that specifically examined gender difference in the prevalence of insomnia as defined by international diagnosis criteria. The findings showed a significantly higher prevalence of insomnia in females (OR = 1.58, 95% CI: 1.35, 1.85), which is higher than the effect size (RR = 1.41, 95% CI: 1.28, 1.55, *p* < 0.001) of the previous meta-analysis (29). This meta-analysis also included recently published studies (8, 10, 18–21, 28, 40–46) and used international diagnostic criteria for insomnia. It should be noted that the previous meta-analysis (29) only included 2 studies using international diagnostic criteria and remaining studies using standardized scales or questions on insomnia, which could increase the heterogeneity caused by measurement instruments. Standardized scales and questions only measure the presence and severity of insomnia symptoms, but cannot establish the diagnosis of insomnia. In addition, RR was inappropriately used as the effect size in the previous meta-analysis (29), therefore the direct comparison between both meta-analyses should be made with caution.

The reasons for the higher prevalence of insomnia in females are multifactorial. Females are more vulnerable to negative socioeconomic factors, such as lower income or education level (47). In addition, females are more likely to experience certain physical problems compared to males, such as osteoporosis, fractures, and back problems (48). Furthermore, females have higher risk of developing certain psychiatric problems, such as depression and anxiety (49–51), all of which could increase the risk of insomnia in females.

Subgroup analyses found that greater gender difference in the prevalence of insomnia was associated with case-control study design and consecutive sampling method. Generally, studies using cohort study design and random sampling could generate better representativeness (52). In addition, a small number of included studies used case-control study design (*n* = 1) and consecutive sampling method (*n* = 1), therefore, their moderating effects on the results need to be further examined. Meta-regression analyses revealed that studies with higher proportion of females and better study quality were significantly associated with greater gender difference in the prevalence of insomnia, which may be due to insomnia being more accurately identified in higher quality studies (52). In addition, since females are more likely to have insomnia (24, 25), studies with higher proportion of females may show a greater gender difference.

The strengths of this meta-analysis are the inclusion of newly published studies and the use of international diagnostic criteria to accurately establish the diagnosis of insomnia. Several limitations need to be acknowledged. First, high heterogeneity was still present in subgroup analyses, because it is difficult to avoid in meta-analysis of observational studies (53). Second, some relevant factors associated with insomnia, such as insomnia type, duration of insomnia, comorbid psychiatric disorders, and marital status, were not reported in most studies, and so were not examined in the subgroup and meta-regression analyses. Finally, only studies conducted in the general population were included, therefore the findings cannot be generalized to special population groups.

In summary, this meta-analysis found that females had significantly higher prevalence of insomnia than males in the general population. Considering the increasing demands on health care services (54) and the negative health effects due to insomnia, preventive measures, regular screening, and effective interventions should be developed and implemented in the general population, especially for females.

## Data Availability Statement

All the data that support the findings of this meta-analysis are available in Tables and Figures.

## Author Contributions

L-NZ, L-GC, and Y-TX: study design and drafting of the manuscript. L-NZ, Q-QZ, YY, LZ, and Y-FX: data collection, analysis, and interpretation. CN: critical revision of the manuscript. All authors: approval of the final version for publication.

## Conflict of Interest

The authors declare that the research was conducted in the absence of any commercial or financial relationships that could be construed as a potential conflict of interest.
