# Supplementary figures and images for: Gender Difference in the Prevalence of Insomnia: A Meta-Analysis of Observational Studies
Source: Front Psychiatry. 2020 Nov 20;11:577429. doi: 10.3389/fpsyt.2020.577429 (PMC7714764; doi:10.3389/fpsyt.2020.577429)

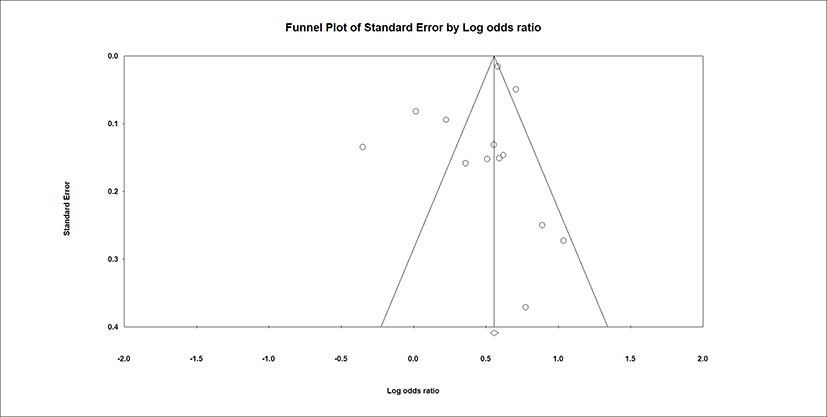

Supplement: Supplementary file 1 [file Image_1.jpg]

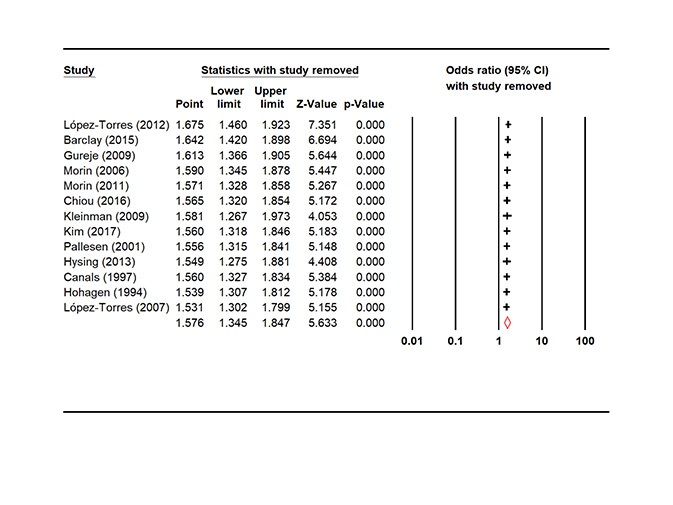

Supplement: Supplementary file 2 [file Image_2.jpg]
